# Detection and Localisation of the Abalone Probiotic *Vibrio midae* SY9 and Its Extracellular Protease, VmproA, within the Digestive Tract of the South African Abalone, *Haliotis midae*

**DOI:** 10.1371/journal.pone.0086623

**Published:** 2014-01-22

**Authors:** Robert J. Huddy, Vernon E. Coyne

**Affiliations:** Department of Molecular and Cell Biology, University of Cape Town, Cape Town, South Africa; University of Central Florida, United States of America

## Abstract

Probiotics have been widely reported to increase the growth rate of commercially important fish and shellfish by enhancing the digestion of ingested feed through the production of extracellular enzymes such as proteases and alginases. In order to investigate this further, the objective of this study was to localise the bacterial probiont *Vibrio midae* SY9 and one of the extracellular proteases it produces in the digestive tract of the South African abalone *Haliotis midae.* This was accomplished by inserting a promotorless *gfp* gene into the chromosome of the bacterium which was incorporated in an artificial, fishmeal-based abalone feed. *In situ* histological comparison of abalone fed either a basal diet or the basal diet supplemented with *V. midae* SY9::Tn10.52 using a cocktail of DNA probes to the *gfp* gene localised the probiont to the crop/stomach and intestinal regions of the *H. midae* digestive tract. Generally, the ingested probiotic bacterium occurred in association with feed and particulate matter within the crop/stomach and intestinal regions, as well as adhered to the wall of the crop/stomach. Histological immunohistochemical examination using polyclonal anti-VmproA antibodies localised an extracellular protease produced by *V. midae* SY9 to the *H. midae* crop/stomach and intestine where it appeared to be associated with feed and/or other particulate matter in the abalone gut. Thus the data suggests that *V. midae* SY9 colonises and/or adheres to the mucous lining of the abalone gut. Furthermore, the close association observed between the bacterium, its extracellular protease and ingested feed particles supports the theory that *V. midae* SY9 elevates *in situ* digestive enzyme levels and thus enhances feed digestion in farmed abalone.

## Introduction

South Africa has a rapidly developing abalone aquaculture industry, based on the cultivation of *Haliotis midae* [1]. However, the relatively slow growth rate of abalone represents a major constraint on the aquaculture industry. The use of probiotic microorganisms is becoming increasingly accepted as a means of improving the health and growth of aquacultured species [2]. Macey and Coyne [3], [4] demonstrated that *H. midae* fed a high protein diet supplemented with the probiotic *Vibrio midae* SY9 had increased digestive tract protease levels, enhanced protein digestion and increased growth rates in comparison to animals fed an un-supplemented diet.

Several possible modes of action have been proposed for probiotic effects observed within aquaculture environments [5], [6], including the production and secretion of extracellular hydrolytic enzymes that contribute to, and improve, the digestion efficiency of the host. Several studies have demonstrated the effect of probiotic supplementation on abalone digestive enzyme activity levels and/or growth, and have suggested a possible role for ‘nutritional probiotics’ in abalone aquaculture [7], [8].

Abalone possess a unique microbiota that is capable of producing extracellular enzymes which degrade the major constituents of abalone feeds [9]. However, less than 10% of the microorganisms associated with the abalone digestive tract can be cultured in the laboratory [10]. Consequently, culture-independent methodologies are necessary for investigating gut microorganisms within their natural habitat [11]. *In situ* hybridization (ISH) using specific 16S rDNA oligonucleotide probes is a culture-independent method used for investigating bacterial population diversity [11], and is an ideal method for investigating microorganisms *in vivo* [12]. ISH techniques have been successfully used to investigate the microbiota of goldfish [13], abalone [4], *Artemia* nauplii [14] and salmon [15], and to specifically localise intracellular prokaryotes in abalone tissue sections [16]. Rengpipat *et al.* [17] successfully tagged the shrimp probiotiont *Bacillus* S11 with GFP and then monitored the presence of this probiotic within the digestive tract of the Black Tiger shrimp *Penaeus monodon* following dietary supplementation. Histological analysis of intestinal samples revealed that the GFP-tagged probiotic bacterium was viable and localised to the surface of the shrimp’s intestine.

Macey and Coyne [3] observed significantly increased growth rates in abalone fed a probiotic supplemented feed, as well as increased protease activity, protein digestion and protein absorption within the intestinal region of these abalone. This finding supports the view that feeding aquacultured species with probiotic microorganism(s) capable of producing and secreting hydrolytic extracellular enzymes may improve digestion efficiency of the host animal, resulting in enhanced host growth rates [18]. Detection of the *V. midae* SY9 extracellular protease VmproA within the digestive tract of *H. midae* fed ABFEED® S34 supplemented with the probiont may indicate that a similar process is responsible for the increased growth rate reported in abalone fed ABFEED® containing the bacterium [3]. Thus the aim of this study was to utilize immunohistochemistry, ISH and standard histological staining techniques to investigate the spatial distribution of *V. midae* SY9 and VmproA within the digestive tract of *H. midae.*

## Materials and Methods

### Ethics Statement

No ethics permits were required for the described study, which complied with all relevant regulations. Permission to work in the Department of Agriculture, Forestry and Fisheries Research Aquarium was not required since it is a research facility that is available to my research group on the basis that my research is government funded. The facility is not attached to a national park. This study did not use any endangered or protected species.

### Microorganisms and Culture Media

The bacterial strains and plasmids used in this study are described in Table 1. *Vibrio midae* SY9 was originally isolated from the gastrointestinal tract of *Haliotis midae* [3]. *V. midae* SY9 was cultured in marine broth (MB) [(wt/vol) 3% NaCl, 0.23% MgCl_2_.6H_2_O, 0.03% KCl, 0.2% glucose, 0.5% casamino acids, 0.1% yeast extract] or peptone marine basal medium (P-MBM) [(wt/vol) 3% NaCl, 0.23% MgCl_2_.6H_2_O, 0.03% KCl, 1% peptone, 0.1% yeast extract] with shaking at 100 rpm at 22°C and maintained on marine agar (MA) [MB supplemented with 2% (wt/vol) bacteriological agar, Unilab] at 22°C. *V. midae* SY9Sm^r^ was grown in VNSS broth [(wt/vol) 1.76% NaCl, 0.147% Na_2_SO_4_, 0.008% NaHCO_3_, 0.025% KCl, 0.004% KBr, 0.187% MgCl_2_.6H_2_O, 0.041% CaCl_2_.2H_2_O, 0.008% SrCl_2_.6H_2_O, 0.008% H_3_BO_3_, 0.1% peptone, 0.05% yeast extract, 0.05% D-glucose, 0.001% FeSO_4_.7H_2_O, 0.001% Na_2_HPO_4_] and maintained on either MA, or on VNSS agar [VNSS broth supplemented with 1.5% (wt/vol) bacteriological agar, Unilab] containing 120 µg/ml streptomycin (Sm) at 22°C, unless otherwise stated. The g*fp* chromosomally-tagged strain, *V. midae* SY9::Tn10.52, was either grown on MA or VNSS agar, or cultivated in P-MBM or MB supplemented with 120 µg/ml Sm and 400 µg/ml kanamycin (Kan) at 22°C unless otherwise stated. *E. coli* SM10λpir harbouring the pLOFKm*gfp* plasmid, obtained from Molecular Biology Vectors (ATCC 87711), was grown at 37°C in either LB10 broth [(wt/vol) 10% NaCl, 10% Tryptone, 5% yeast extract] or on LA10 agar [LB10 broth supplemented with 1.5% (wt/vol) bacteriological agar, Unilab] supplemented with 100 µg/ml Kan and 100 µg/ml ampicillin (Amp).

**Table 1:** Strains and plasmid used in this study.

Strains/Plasmid	Genotype/Relevant characteristic(s)^a^	Reference
Strains:		
*V. midae* SY9	Isolated from the digestive tract of *H. midae*, South Africa	[3]
*V. midae* SY9Sm^r^	Streptomycin resistant strain of *V. midae* SY9; Sm^r^	This study
*V. midae* SY9::Tn10.52	*V. midae* SY9Sm^r^::mini-Tn10-*gfp*-*kan*, Sm^r^, Km^r^	This study
*E. coli* SM10λpir	*thi thr leu ton*A *lac*Y *sup*E (λpir) *rec*A::RP4-2-Tc::Mu Km^r^	[30]
*E. coli* JM109	*recA*1 *supE*44 *endA*1 *hsdR*17 *gyrA*96 *relA*1 *thi*Δ (*lac-proAB*)F’(traD36 proAB’ lacI^q^ lacZΔM15)	[31]
Plasmid:		
pLOFKm*gfp*	pLOFKm with cloned promoterless *gfp* (GFPmut2), Km^r^, Amp^r^	[29]

a Sm^r^, streptomycin resistant; Km^r^, kanamycin resistant; Amp^r^, ampicillin resistant.

### Transposon Mutagenesis and Chromosomal-tagging of *V. midae* SY9

**Figure 1:**
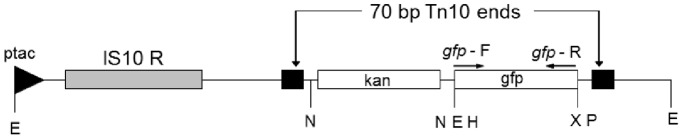
Diagrammatic representation of the mini-Tn10-*gfp*-*kan* transposable element in the recombinant transposon vector pLOFKm*gfp*. The transposase gene of IS10_R_ is located outside the inverted repeats (black-boxes) of the mobile element and downstream of the p*tac* promoter (black triangle). The PCR primers (*gfp*-F and *gfp*-R) are indicated above the open boxes depicting the kanamycin (kan) and *gfp* genes. Relevant restriction enzyme sites are indicated. Abbreviations: E, *Eco*RI; H, *Hin*dIII; M, *Mlu*I; N, *Not*I; P, *Pst*I; S, *Sal*I; Sf, *Sfi*I; X, *Xba*I. The diagram (not to scale) of mini-Tn10-*gfp*-*kan* was adapted from Stretton *et* *al*. [29].

A *V. midae* SY9 strain capable of growth on streptomycin was generated as described by Macey and Coyne [4] and designated *V*. *midae* SY9Sm^r^. The vector pLofKm*gfp* (Table 1), harbouring a mini-Tn10-*gfp*-*kan* transposable element (Fig. 1), was conjugated from *E. coli* SM10λpir into *V. midae* SY9Sm^r^ using a modified filter mating technique [19]. Briefly, 5 ml cultures of *E. coli* SM10λpir harbouring pLofKm*gfp* and *V. midae* SY9Sm^r^ were cultivated for 16 hours. The donor and recipient cultures were gently mixed at a volume ratio of 1∶10 (100 µl donor and 1000 µl recipient) in 5 ml of wash solution [50% (vol/vol) Nine Salts Solution (NSS) (wt/vol): 0.88% NaCl, 0.0735% Na_2_SO_4_, 0.004% NaHCO_3_, 0.0125% KCl, 0.002% KBr, 0.0935% MgCl_2_.6H_2_O, 0.0205% CaCl_2_.2H_2_O, 0.004% SrCl_2_.6H_2_O, 0.004% H_3_BO_3_∶50% (vol/vol) 10 mM MgSO_4_]. The mixture was passed through a 0.22 µm filter (Whatman) and washed with another 5 ml of wash solution, before being placed cell-side up on LA-20 agar [(wt/vol) 20% NaCl, 10% Tryptone, 5% yeast extract, and 1.5% (wt/vol) bacteriological agar, Unilab] and incubated at 30°C for 4 hours. The filter discs were then placed inside sterile 50 ml plastic tubes (Sterilin) containing 2 ml of NSS and vortexed vigorously to resuspend the bacteria which were spread-plated on VNSS agar containing 120 µg/ml Sm and 400 µg/ml Kan. Aliquots of each donor and recipient strain were spread-plated separately on VNSS agar and VNSS agar containing 120 µg/ml Sm and 400 µg/ml Kan as mating controls.

### Screening Transconjugants

*V. midae* SY9 strains putatively harbouring the chromosomally integrated mini-Tn10-*gfp*-*kan* transposable element were inoculated into 5 ml MB containing 120 µg/ml Sm and 400 µg/ml Kan, and grown for approximately 16 hours at 22°C on a rotary shaker. Chromosomal DNA was isolated according to the method described by Ausubel *et al.* [20]. PCR amplification of the *gfp* gene using the oligonucleotide primers *gfp*-F (5′-GATTTCTAGATTTAAGAAGC-3′) and *gfp*-R (5′-TCATATTTGTATAGTTCATCC-3′) was used to screen for the presence of the integrated *gfp* gene in order to identify *gfp*-tagged *V. midae* SY9::Tn10.52 strains for further analysis.

### Southern Hybridization

The plasmid pLOFKm*gfp* was purified from *E. coli* SM10λpir using the Qiagen Midi Prep kit according the manufacturer’s instructions. A 717 bp DNA fragment of the *gfp* gene was PCR amplified from pLOFKm*gfp* using the synthetic oligonucleotide primers *gfp*-F and *gfp*-R. The 717 bp PCR amplicon was resolved on a 1% (wt/vol) TAE agarose gel and purified using the BioSpin Gel Extraction Kit (BioFlux) according to the manufacturer’s instructions. The purified 717 bp DNA fragment was radioactively labelled using a random-primed DNA labelling kit (Roche) according to the manufacturer’s instructions and hybridised to equal amounts of *V. midae* SY9 and *V. midae* SY9::Tn10.52 chromosomal DNA. The Southern hybridization procedure was performed according to Church and Gilbert [21].

### Probe Design and Labelling

Three oligonucleotide probes GFP001 (5′-X-GTTGAATTAGATGGTGATGTTAATGG-3′, where X denotes the DIG label), GFP002 (5′-CTACCTGTTCCATGGCCAACACTTG-3′) and GFP003 (5′-CAAAATACTCCAATTGGCGATGGCCCTG-3′) were designed to hybridize to segments of the *gfp* gene of pLOFKm*gfp*. The 16S rRNA gene eubacterial probe EUB338 (5′-GCTGCCTCCCGTAGGAGT-3′) [22] was employed as an *in situ* hybridization control. The probe ECJ109 (5′-GAGTAAAGTTAATACCTTTGCTCA-3′) was used as a negative control of the *in situ* hybridization experiments. All of the oligonucleotide probes were 5′-DIG labelled (Roche) under weak alkaline conditions in a sodium borate buffer/dimethyl formamide mixture at 22°C overnight [23].

### Preparation of *V. midae* SY9::Tn10.52-supplemented Feed

The basal diet consisted of ABFEED® S34 weaning chips as supplied by Marifeed (Pty.) Ltd., Hermanus, South Africa. The *gfp* chromosomally-tagged probiotic strain *V. midae* SY9::Tn10.52 was incorporated into ABFEED® S34 weaning chips by vacuum infusion, to a final concentration of at least 1.0×10^8^ culturable cells g^-1^ feed. Briefly, *V. midae* SY9::Tn10.52 was cultivated for 24 hrs in 3 litres of P-MBM supplemented with 120 µg/ml Sm and 400 µg/ml Kan on an orbital shaker (100 rpm) at 22°C. Thereafter, the bacterial cells were harvested by centrifugation (8,000×g for 15 minutes at 4°C), washed with one volume of artificial seawater (ASW) [(wt/vol): 3% NaCl, 0.23% MgCl_2_.6H_2_O, 0.03% KCl] and resuspended in 100 ml of ASW. Approximately 200 g of the ABFEED® S34 weaning chips were sealed inside a glass vacuum jar and a vacuum drawn within the jar to approximately 80 kPa. Approximately 20 ml of the *V. midae* SY9::Tn10.52 suspension was drawn into the vacuum jar and thoroughly mixed with the feed. During the process of drawing the bacterial suspension into the chamber, the vacuum was decreased to 50 kPa and maintained at this pressure for 5 minutes. Thereafter, the vacuum was slowly released, the impregnated ABFEED® S34 weaning chips removed from the jar and dried at 22°C overnight, before being sealed into clean plastic bags and stored at 4°C. Fresh batches of feed were prepared every 7 days and each batch was analyzed by determining the total number of culturable bacterial cells to ensure that there was at least 1×10^6^ culturable *V. midae* SY9::Tn10.52 cells g/feed.

### Animals

Juvenile *H. midae* (<15 mm shell length) were kept at the Department of Agriculture, Fisheries and Forestry Research Aquarium (Cape Town, South Africa). Abalone were maintained in plastic mesh baskets (each containing approximately 50 animals) in adjacent 30 litre tanks supplied with aerated and continuously flowing (approximately 100 l/hr) sand-filtered sea water at 15–18°C, and subjected to a 10∶14 (light:dark) light-cycle. The animals were acclimatized for 3 weeks prior to the start of the experiment. During acclimatization the abalone were fed ABFEED® S34 weaning chips to satiation. All uneaten food was removed from the baskets and the tanks and baskets were thoroughly cleaned every 2 days before the addition of fresh feed.

Abalone in both tanks were starved for a period of 24 hours prior to the beginning of the experiment. At the start of the experiment (Day 0), six randomly selected animals were removed from each tank and immediately sacrificed. The remaining abalone in one tank were fed the *V. midae* SY9::Tn10.52-supplemented diet, while those in the other tank were fed the basal diet. The animals were fed to satiation on the respective diets for the duration of the experiment. Six randomly selected animals were sacrificed from each treatment group on days 2 and 14.

### Histology

The abalone shell was gently removed by severing the adductor muscle as close to the shell as possible using a thin metal spatula, without rupturing the digestive tract. The animals were placed adductor muscle side down in labeled embedding cassettes and fixed in Davidson’s solution [24] (per litre (vol/vol): 330 ml 95% ethanol (Merck), 220 ml 100% formalin (Merck), 115 ml glacial acetic acid (Merck) and 335 ml distilled water) for 36 hours at 4°C, before being transferred to 70% (vol/vol) ethanol at 4°C. Following fixation, the abalone samples were dehydrated through a graded ethanol series to 100% xylene (Saarchem) in a Tissue Trek II tissue processor. The dehydrated tissue samples were embedded in paraffin wax, sectioned at 5 µm, adhered onto positively charged microscope slides (SuperFrost® Plus, Menzel-Gläser) and stored in slide boxes at room temperature.

The *H. midae* sections were deparaffinised and stained using standard Harris’ H&E stain according to Hayat [25] and mounted with phosphate-buffered glycerin jelly [50% (vol/vol) Glycerol (Merck), 0.5 M phosphate buffer (pH 7.0) and 7.5% (wt/vol) gelatine (Merck)]. The sections were viewed using a Nikon Eclipse 50 i Compound Microscope equipped with a Nikon DS Camera Control Unit DS-U2 and DS-5M Camera head with Nikon Software (NIS Elements Documentation and Digital 3D Imaging).

### In situ Hybridization

*H. midae* sections were processed for ISH after being deparaffinized and rehydrated through an ethanol series [99.9, 95, 80, 70, and 50% (v/v) ethanol]. Slides were covered with pre-warmed ISH buffer [50% (vol/vol) formamide (Sigma), 4x SSC, 1x Denhardt’s solution (Sigma), 0.2 mg yeast tRNA (Sigma), 0.5 mg denatured Herring sperm DNA (Sigma)] and pre-hybridized at 42°C for 60 minutes in a humid chamber to reduce non-specific hybridization of the probe(s). Thereafter, the tissue sections were heat denatured on a heating block (98°C for 10 minutes) and subsequently cooled rapidly on ice. The sections were probed with the cocktail of *gfp*-specific probes (GFP001, GFP002 and GFP003). Each probe was added at a concentration of approximately 6.66 pmol/ml of ISH buffer, resulting in a total probe concentration of 20 pmol/ml. The positive (EUB338) and the negative (ECJ109) control oligonucleotide probes were added at a concentration of 20 pmol/ml of ISH buffer.

Pre-heated ISH buffer containing 20 pmol/ml of the DIG-labeled probes was evenly layered onto the tissue sections and incubated for approximately 16 hours at 40°C in a humid chamber. Any unbound DIG-labeled probes were removed from the tissue sections by washing twice for 5 minutes in 2x SSC at 22°C with gentle agitation, twice for 5 minutes in 1x SSC at 22°C with gentle agitation, and twice for 10 minutes in 0.5x SSC at 40°C with gentle agitation. The tissue sections were then equilibrated in buffer 1 [150 mM NaCl, 100 mM Tris-HCl (pH 7.5)] for 2 minutes at 22°C. The sections were washed in blocking buffer [100 mM Tris-HCl (pH 7.5), 150 mM NaCl, 2% (vol/vol) feotal calf serum (Invitrogen) and 0.3% (vol/vol) Triton X-100 (Sigma)] for 60 minutes at 22°C with gentle agitation to reduce non-specific antibody binding. Anti-digoxigenin-AP (Roche) signalling molecule was applied to the sections which were incubated for 3 hrs at 22°C in a humid chamber.

The tissue sections were rinsed briefly with buffers 1 and 2 [100 mM Tris-HCl (pH 9.5), 100 mM NaCl, 50 mM MgCl_2_], respectively, before being overlaid with a NBT/BCIP (Roche) colour development solution, prepared in buffer 2 according to the manufacturer’s instructions, and incubated at 22°C in a dark humid chamber until a signal was visible. The colour development reaction was stopped by washing the slides with TE buffer [10 mM Tris-HCl (pH 7.6), 2 mM EDTA] and rinsing with distilled water. The sections were counter-stained with 0.05% (w/v) aqueous Methyl Green (Sigma) for 2 minutes to enhance the background tissue morphology, rinsed with dH_2_O, dried and mounted as described above. Hybridization signals indicating the presence of the target microorganism(s) were visible as a purple precipitate where the DIG-labelled probe had bound to homologous target DNA within the tissue sections. The stained and mounted sections were viewed and photographed as described above.

### Immunohistochemical Localisation of VmproA

Abalone tissue sections were prepared and deparaffinized as described above, and subsequently pre-hybridized with immunohistochemistry buffer [1x PBS (pH 7.4), 5% (vol/vol) fetal calf serum, 1% (vol/vol) DMSO (Sigma), 0.2% (wt/vol) BSA (Roche), and 0.1% (vol/vol) Tween-20 (Saarchem)] at 22°C for 2 hours in a humid chamber. The pre-hybridization solution was replaced with fresh immunohistochemistry buffer containing anti-[VmproA] polyclonal antibodies that had been pre-absorbed against *E. coli* JM109 cellular extract and abalone tissue powder according to [26] and the sections incubated in a humid chamber for 24 hours at 4°C. The slides were briefly rinsed with PBT [1x PBS (pH 7.4) and 0.2% (vol/vol) Tween-20] at 22°C with gentle agitation to remove unbound anti-[VmproA] antibodies. Thereafter, the tissue sections were incubated with fresh immunohistochemistry buffer containing goat anti-[rabbit AP-conjugated] secondary antibodies (Sigma) diluted 1∶1000 for 3 hours at 22°C in a humid chamber. Unbound secondary antibodies were removed by sequential washes in PBT at 22°C with gentle agitation.

The tissue sections were rinsed briefly with buffer 1 and buffer 2, respectively, overlaid with NBT/BCIP (Roche) colour development solution and incubated at 22°C in a dark humid chamber until a signal was clearly visible. The colour development reaction was stopped by washing the slides with TE buffer and rinsing with distilled water, and the slides treated and mounted as described above. AP-conjugated anti-rabbit secondary antibodies bound to anti-[VmproA] polyclonal antibodies that had in turn bound to VmproA within the tissue sections were visible as areas of purple precipitate (NBT/BCIP). The stained and mounted sections were viewed and photographed as described above.

## Results

### Transposon Mutagenesis of *V. midae* SY9

Fifty eight *V. midae* SY9Sm^r^ cells growing on VNSS agar supplemented with Sm and Kan were screened by PCR amplification in order to confirm chromosomal integration of the mini-Tn10-*gfp*-kan cassette (data not shown). Three strains were identified as being *gfp* chromosomally-tagged *V. midae* SY9 strains, one of which, designated *V. midae* SY9::Tn10.52, was selected for further characterisation.

### Southern Hybridization

**Figure 2:**
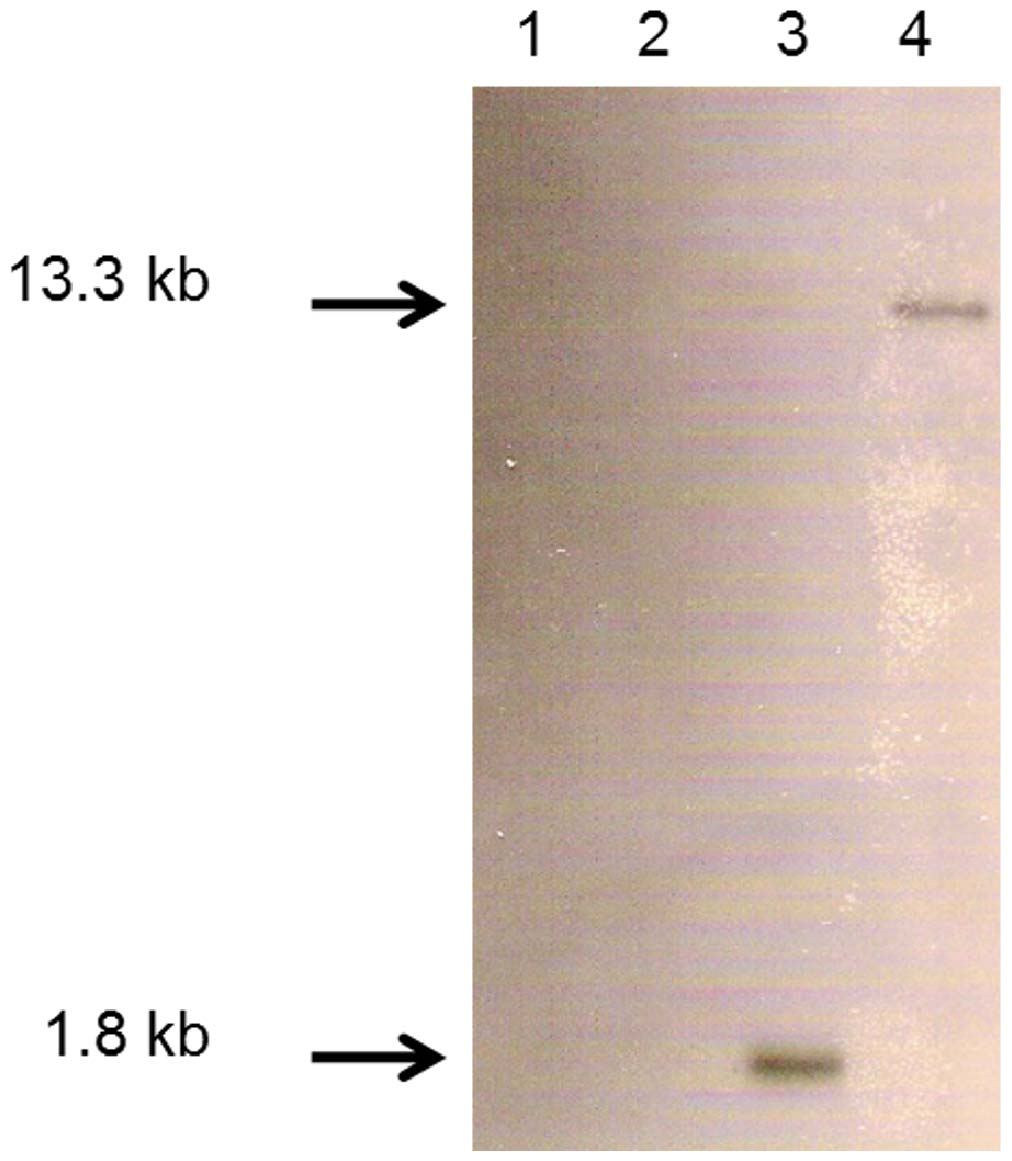
Southern hybridisation of *V. midae* SY9 and *V. midae* SY9::Tn10.52 chromosomal DNA showing single-site integration of the mini-Tn10-*gfp*-*kan* transposable element in the genome of *V. midae* SY9Sm^r^. *V. midae* SY9 chromosomal DNA digested with *Hind*III (lane 1) and *Eco*RI (lane 2), and *V. midae* SY9::Tn10.52 chromosomal DNA digested with *Hin*dIII (lane 3) and *Eco*RI (lane 4). The arrows indicate the approximate sizes of the bands in kilobase pairs (kb).

Mini-Tn10-gfp-kan integration into the *V. midae* SY9 genome was confirmed by a Southern hybridisation experiment using a 0.7 kb fragment of the *gfp* gene as a probe against chromosomal DNA isolated from *V. midae* SY9 and *V. midae* SY9::Tn10.52 (Fig. 2). The presence of a single hybridisation band of 1.8 and 13.3 kb following hybridisation of the *gfp* fragment to *Hin*dIII- and *Eco*RI-digested *V. midae* SY9::Tn10.52 chromosomal DNA indicated that the transposable element integrated at a single site in the *V. midae* SY9 genome.

### Histology

Morphological differences between *H. midae* fed either the ABFEED® S34 basal diet or the *V. midae* SY9::Tn10.52 supplemented feed were not observed by histological examination of H&E stained whole-animal sections over the course of the 14 day experimental period (data not shown). The oesophageal region of the alimentary canal was devoid of any food in both groups of animals, while the crop/stomach and intestinal regions contained mucous and food particles.

### Localization of *V. midae* SY9 within the *H. midae* Digestive Tract

**Figure 3:**
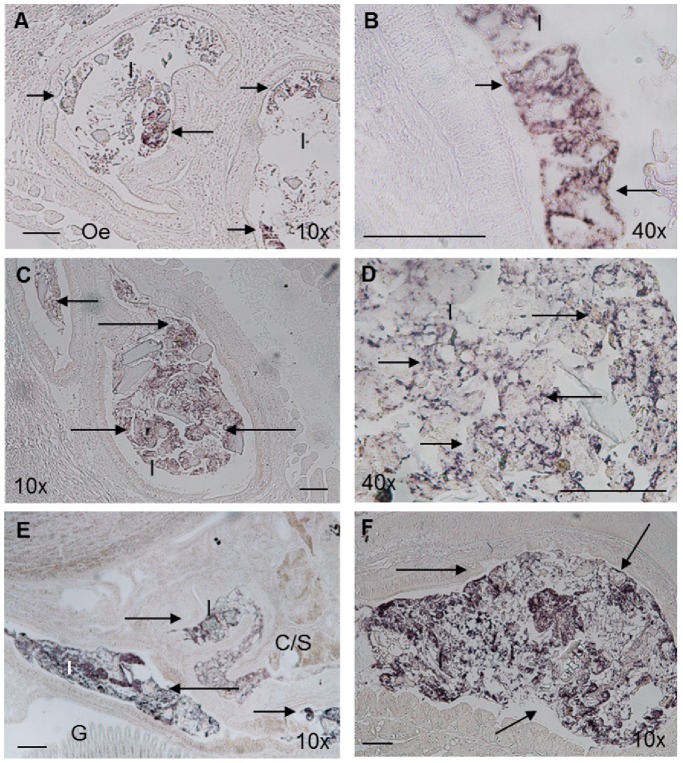
*In situ* hybridization of whole-*H. midae* tissue sections with the universal eubacterial probe EUB338, 0 (A and B), 2 (C and D) and 14 days (E and F) of feeding basal ABFEED® S34. Arrows indicate eubacteria associated with food in the lumen of the intestine. Scale bar, 100 µm. Magnification indicated on each panel. Abbreviations used: C/S, Crop/stomach; I, Intestine; G, Gills; Oe, Oesophagus.

**Figure 4:**
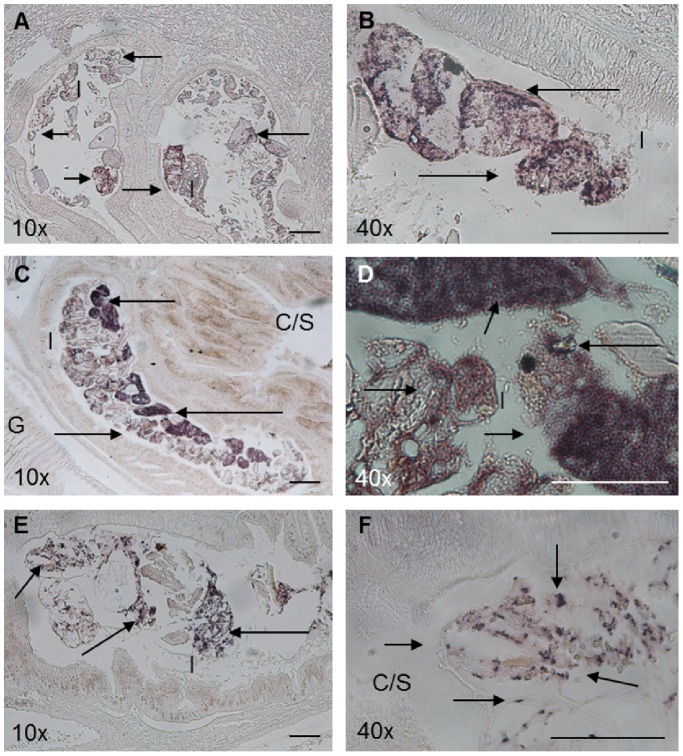
*In situ* hybridization of whole-*H. midae* tissue sections with the universal eubacterial probe EUB338, 0 (A and B), 2 (C and D) and 14 days (E and F) of feeding *V. midae* SY9::Tn10.52-supplemented ABFEED® S34. Arrows indicate eubacteria associated with food in the lumen of the crop/stomach (F) and intestine (A-E). Scale bar, 100 µm. Magnification indicated on each panel. Abbreviations used: C/S, Crop/stomach; I, Intestine; G, Gills.

**Figure 5:**
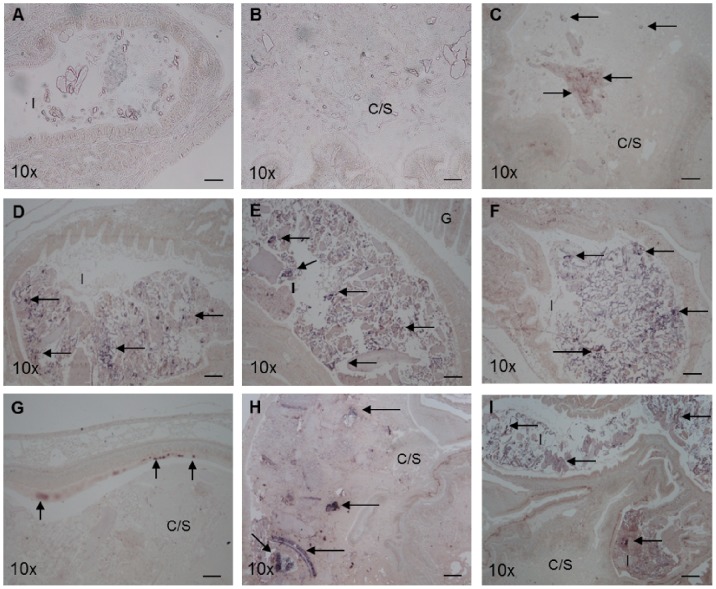
*In situ* hybridization of whole-*H. midae* tissue sections with a cocktail of *gfp*-specific oligonucleotide probes GFP001, GFP002 and GFP003, 0 (A and B), 2 (C, D and E) and 14 days (F, G, H and I) of receiving a *V. midae* SY9::Tn10.52-supplemented ABFEED® S34 diet. Panels A (intestinal lumen) and B (lumen of the crop/stomach) lack hybridization signals since the animals were sampled at the start of the experiment (Time = 0). Arrows indicate GFP-tagged *Vibrio midae* SY9 associated with food in the lumen of the crop/stomach (C and H) and intestine (D – F, I). Panel G depicts GFP-tagged *V. midae* SY9 associated with the epithelial lining of the crop/stomach. Scale bar, 100 µm. Magnification indicated on each panel. Abbreviations used: C/S, Crop/stomach; I, Intestine; G, Gills.

ISH was used to detect the presence of *V. midae* SY9 in the digestive tract of *H. midae* fed ABFEED® S34 supplemented with *V. midae* SY9::Tn10.52 over a fourteen day period. Strong hybridization signals were observed within the crop/stomach and intestinal regions of *H. midae* fed either the basal or the *V. midae* SY9::Tn10.52 -supplemented feeds when whole-animal *H. midae* tissue sections were hybridized with the universal eubacterial probe EUB338 (Figures 3 and 4). The signals appear to be associated with food and particulate matter in the crop/stomach and intestine. Hybridization signals were not detected within the oesophageal or digestive gland regions of the *H. midae* digestive tract (data not shown). Hybridization signals were not detected in whole-animal sections prepared from abalone fed the basal ABFEED® S34 diet for 0, 2 or 14 days probed with the *gfp*-specific oligonucleotide cocktail (data not shown). Similarly, the *gfp*-specific probes did not produce detectable hybridization signals in sections prepared from abalone fed the *V. midae* SY9::Tn10.52 supplemented diet sampled at day 0 (Fig. 5). However, hybridization signals were detected in the crop/stomach and intestine of whole-animal sections of *H. midae* fed *V. midae* SY9::Tn10.52 supplemented ABFEED® S34 for 2 and 14 days (Fig. 5). The hybridization signals appear to be associated with feed and/or particulate matter in these regions of the abalone digestive tract, as well as along the lining of the crop/stomach. The absence of hybridization signals in whole-animal *H. midae* sections hybridized with the *E. coli* JM109-specific DIG-labelled oligonucleotide probe ECJ109 confirmed the specificity of the signals obtained with the universal eubacterial probe and the cocktail of *gfp*-specific oligonucleotide probes (data not shown).

### Immunohistochemical Localization of VmproA within the *H. midae* Digestive Tract

**Figure 6:**
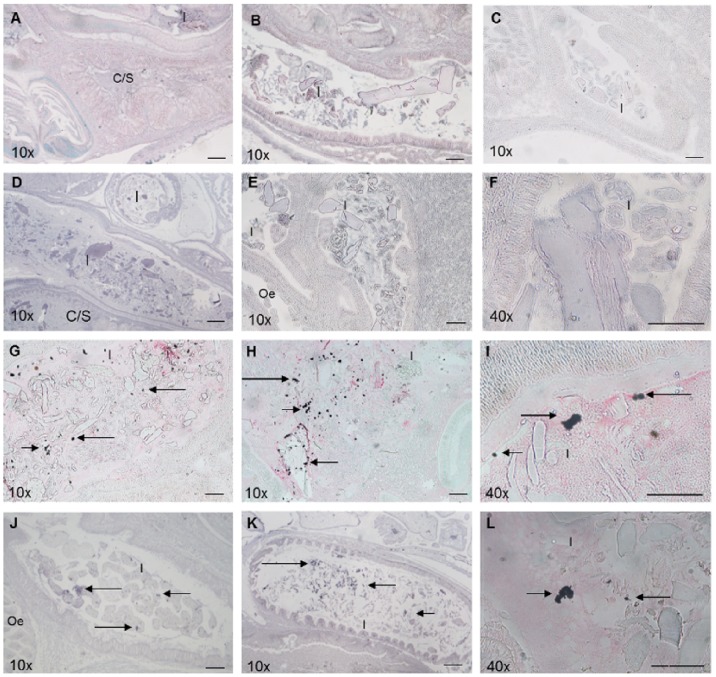
Immunohistochemical detection of VmproA within the digestive tract of whole-*H. midae* sections 0 (A-B), 2 (C) and 14 (D) days after receiving basal ABFEED®S34 and 0 (E-F), 2 (G-I) and 14 (J-L) days after receiving a *V. midae* SY9::Tn10.52-supplemented ABFEED® diet. Panels A (crop/stomach) and B-D (intestinal lumen) lack positive signal since the animals were fed unsupplemented ABFEED®. Panels E (portion of the oesophagus and intestinal lumen) and F (lumen of the intestine) lack positive signals since the animals, although fed a *V. midae* SY9::Tn10.52-supplemented ABFEED® diet, were sampled at the start of the experiment (Time = 0). Arrows indicate VmproA associated with food in the lumen of the intestine of abalone fed the *V. midae* SY9::Tn10.52-supplemented ABFEED® diet (G - L). Scale bar, 100 µm. Magnification indicated on each panel. Abbreviations used: I, Intestine; C/S, Crop/stomach; Oe, Oesophagus.

Immunohistochemical signals corresponding to the presence of anti-VmproA polyclonal antibodies were detected in the crop/stomach and intestinal regions of whole-animal sections prepared from *H. midae* fed ABFEED® S34 supplemented with *V. midae* SY9::Tn10.52 (Fig. 6). VmproA appears to be associated with food and/or other particulate matter within both the crop/stomach and intestine. Immunostaining signals were not evident in whole-animal sections prepared from *H. midae* fed unsupplemented ABFEED® S34 (Fig. 6).

## Discussion

Macey and Coyne (2005) investigated *in situ* protease activity and protein digestion in *H. midae* fed a high-protein artificial diet supplemented with a mixture of three probiotic strains that included *V. midae* SY9. They found that *H. midae* fed ABFEED® S34 supplemented with the probiotic strains had enhanced intestinal alkaline protease activity in comparison to animals fed a basal ABFEED® S34 diet. They also observed a significant improvement in intestinal protein digestion and absorption in the abalone fed the probiotic-supplemented diet [3].

Rengpipat *et al.* [17] transformed *Bacillus* S11 with the GFP-expressing plasmid pAD44-12 which they used as a marker for fluorescent *in situ* localisation of *Bacillus* S11 in the intestine of the black tiger shrimp *Penaeus monodon*. Similarly, Macey and Coyne [4] constructed chromosomally tagged strains of *V. midae* SY9 using a mini-Tn*10*-*gfp*-*kan* transposon mutagenesis system in order to track the persistence of ingested *V. midae* SY9 within the digestive tract of *H. midae*. However, Macey [3] observed that relative GFP expression and fluorescence in the *V. midae* SY9 mini-Tn*10*-*gfp*-*kan* transconjugants was inadequate for *in situ* detection of *V. midae* SY9 in the abalone digestive tract. Nevertheless, Macey and Coyne [4] demonstrated that a fragment of the mini-Tn*10*-*gfp*-*kan* transposon cassette could be used as a specific DNA probe to detect chromosomally tagged *V. midae* SY9 in the digestive tract of *H. midae*. Chromosomal integration of the mini-Tn*10*-*gfp*-*kan* transposon had no significant impact on the growth and protease activity of the *gfp* chromosomally-tagged *V. midae* SY9 strains (data not shown). Similarly, Rengpipat *et al.* [17] demonstrated that the properties of a GFP-expressing mutant strain of a shrimp probiont *Bacillus* S11-GFP were not significantly different to that of the wild-type strain. Therefore, *in situ* hybridization (ISH) using specific DNA probes was used in this study to localise ingested *V. midae* SY9 in the *H. midae* digestive tract.

Bacteria are known to occur throughout the digestive tract of aquatic invertebrates [27]. Previous studies have identified a variety of bacterial isolates from the abalone gut [3], [18], [28]. Therefore, it is not surprising that, regardless of diet, strong hybridization signals were detected in both the crop/stomach and intestinal regions when whole-animal sections were probed with the 16S rRNA gene eubacterial probe EUB338. [18] demonstrated that comparatively, the intestine contained the largest number and greatest diversity of culturable enteric bacteria in the *H. midae* digestive system. Similarly, hybridization signals associated with the intestinal region of the digestive tract appeared to be more intense than those observed in the crop/stomach, indicating a greater load of enteric bacteria within the posterior regions of the abalone digestive tract.

*In situ* hybridization of whole-animal tissue sections using a cocktail of *gfp*-specific oligonucleotide probes detected *V. midae* SY9::Tn10.52 in the crop/stomach and intestinal regions of the *H. midae* digestive tract. In contrast, hybridization signals were not detected in sections prepared from abalone fed the basal ABFEED® S34 weaning chips. Similar results have been reported in studies investigating the colonisation potential of probiotics for shrimp [17] and abalone [4]. Macey and Coyne [4] used cell culture and *in situ* hybridization to detect the chromosomally-tagged strain *V. midae* SY9.8 in both the crop/stomach and intestine of *H. midae* fed probiotic-supplemented feed.

We observed distinct hybridization signals indicating the presence of *V. midae* SY9::Tn10.52 along the inner surface of the crop/stomach of *H. midae* fed supplemented ABFEED®. Similarly, Rengpipat *et al.* [17] detected GFP-tagged *Bacillus* S11 (S11-GFP) cells attached to the intestinal mucous lining of the black tiger shrimp *P. monodon*. Additionally, strong hybridization signals were observed in association with feed or other particulate matter within the crop/stomach and intestine of *H. midae* fed the *V. midae* SY9::Tn10.52 supplemented diet.

The extracellular protease VmproA produced by *V. midae* SY9 was detected immunologically within the crop/stomach and intestinal regions of *H. midae* fed ABFEED® S34 supplemented with *V. midae* SY9::Tn10.52. There was no immunohistochemical evidence of VmproA within the *H. midae* digestive tract of abalone fed the basal diet over the course of the 14 day experimental period. VmproA appeared to be less abundant in the crop/stomach than the intestine of abalone fed *V. midae* SY9::Tn10.52 supplemented feed. Similarly, elevated levels of *in situ* alkaline protease activity detected in the crop/stomach of abalone fed ABFEED® supplemented with *V. midae* SY9 were not as pronounced as the protease activity observed in the intestinal regions of these animals [4]. Since *V. midae* SY9::Tn10.52 cells detected in the intestine by *in situ* hybridization with the *gfp*-specific probes was more abundant in comparison to the crop/stomach, it is likely that the relative abundance of detectable VmproA in the intestine is a function of the elevated *V. midae* SY9 cell number in this portion of the *H. midae* digestive tract. Indeed, a significant positive correlation (*r* = 0.711, *P*<0.05, n = 17) was found between intestinal protease activity and the number of *V. midae* SY9.8 cells present, supporting an association between *V. midae* SY9 and protease activity in the intestine of *H. midae* [4].

VmproA appeared to be generally associated with the feed and/or particulate matter within the intestine of *H. midae* fed the *V. midae* SY9::Tn10.52 -supplemented ABFEED® diet. Immunohistochemical localisation of VmproA within the *H. midae* digestive tract appears to be similar to the *in situ* localisation of *V. midae* SY9::Tn10.52 detected by the *in situ* hybridization analysis of whole-animal tissue sections. Thus, it could be hypothesised that viable *V. midae* SY9 cells, ingested with the supplemented ABFEED® pellets, may attach to the surface of the digestive tract but are mostly associated with food particles and/or other particulate matter in the abalone digestive tract. Presumably, proximity to protein-rich ABFEED® induces *V. midae* SY9 to secrete proteases such as VmproA within the digestive tract of *H. midae*.

## Conclusion

This study demonstrated that *V. midae* SY9 and its extracellular protease, VmproA, could be localised within the digestive tract of *H. midae* fed a *V. midae* SY9-supplemented diet. The data suggests that *V. midae* SY9 may colonise and/or adhere to the mucous lining of the abalone gut, as well as associate with the surfaces of ingested food particles passing through the digestive tract. In so doing, *V. midae* SY9 may elevate *in situ* enzyme levels and thus enhance feed digestion. To the best of our knowledge this is the first study to localize an extracellular protease produced by a probiotic bacterium in the abalone digestive tract.

## References

[1] Troell M, Anderson RJ, Bolton JJ, Maneveldt G, Halling C, et al (2006) Abalone farming in South Africa: An overview with perspectives on kelp resources, abalone feed, potential for on-farm seaweed production and socio-economic importance. Aquaculture 257: 266–281.

[2] Gomez-gil B, Roque A, Turnbull JF (2000) The use and selection of probiotic bacteria for use in the culture of larval aquatic organisms. Aquaculture 191: 259–270.

[3] Macey BM, Coyne VE (2005) Improved growth rate and disease resistance in farmed *Haliotis midae* through probiotic treatment. Aquaculture 245: 249–261.

[4] Macey BM, Coyne VE (2006) Colonization of the gastrointestinal tract of the farmed South African abalone *Haliotis midae* by the probionts *Vibrio midae* SY9, *Cryptococcus* sp. SS1, and *Debaryomyces hansenii* AY1. Marine Biotechnology (New York, NY) 8: 246–259. 10.1007/s10126-005-0113-9 16532366

[5] Verschuere L, Rombaut G, Sorgeloos P, Verstraete W (2000) Probiotic bacteria as biological control agents in aquaculture. Microbiology and Molecular Biology Reviews 64: 655–671. 11104813 10.1128/mmbr.64.4.655-671.2000 PMC99008

[6] Balcázar JL, Blas I de, Ruiz-Zarzuela I, Cunningham D, Vendrell D, et al (2006) The role of probiotics in aquaculture. Veterinary Microbiology 114: 173–186. 16490324 10.1016/j.vetmic.2006.01.009

[7] ten Doeschate KI, Coyne VE (2008) Improved growth rate in farmed *Haliotis midae* through probiotic treatment. Aquaculture 284: 174–179.

[8] Iehata S, Inagaki T, Okunishi S, Nakano M, Tanaka R, et al (2010) Improved gut environment of abalone *Haliotis gigantea* through *Pediococcus* sp. Ab1 treatment. Aquaculture 305: 59–65.

[9] Sawabe T, Oda Y, Shiomi Y, Ezura Y (1995) Alginate degradation by bacteria isolated from the gut of sea urchins and abalones. Microbial Ecology 30: 193–202. 24185485 10.1007/BF00172574

[10] Tanaka R, Sugimura I, Sawabe T, Yoshimizu M, Ezura Y (2003) Gut microflora of abalone *Haliotis discus hannai* in culture changes coincident with a change in diet. Fisheries Science 69: 951–958.

[11] Tanaka R, Ootsubo M, Sawabe T, Ezura Y, Tajima K (2004) Biodiversity and *in situ* abundance of gut microflora of abalone (*Haliotis discus hannai*) determined by culture-independent techniques. Aquaculture 241: 453–463.

[12] Moter A, Göbel UB (2000) Fluorescence *in situ* hybridization (FISH) for direct visualization of microorganisms. Journal of Microbiological Methods 41: 85–112. 10991623 10.1016/s0167-7012(00)00152-4

[13] Asfie M, Yoshijima T, Sugita H (2003) Characterization of the goldfish fecal microflora by the fluorescent *in situ* hybridization method. Fisheries Science 69: 21–26.

[14] Høj L, Bourne DG, Hall MR (2009) Localization, abundance and community structure of bacteria associated with *Artemia*: Effects of nauplii enrichment and antimicrobial treatment. Aquaculture 293: 278–285.

[15] Holben W, Williams P, Saarinen M, Särkilahti L, Apajalahti J (2002) Phylogenetic analysis of intestinal microflora indicates a novel *Mycoplasma* phylotype in farmed and wild salmon. Microbial Ecology 44: 175–185. 12082453 10.1007/s00248-002-1011-6

[16] Antonio DB, Andree KB, Moore JD, Friedman CS, Hedrick RP (2000) Detection of Rickettsiales-like prokaryotes by in situ hybridization in black abalone, *Haliotis cracherodii*, with withering syndrome. Journal of Invertebrate Pathology 75: 180–182. 10772333 10.1006/jipa.1999.4906

[17] Rengpipat S, Wongtangprasert N, Palaga T (2009) The use of green fluorescent protein as a marker for monitoring a probiotic *Bacillus* S11 in the black tiger shrimp *Penaeus monodon* . Aquaculture Nutrition 15: 297–305.

[18] Erasmus JH, Cook PA, Coyne VE (1997) The role of bacteria in the digestion of seaweed by the abalone *Haliotis midae* . Aquaculture 155: 377–386.

[19] Egan S, James S, Kjelleberg S (2002) Identification and characterization of a putative transcriptional regulator controlling the expression of fouling inhibitors in *Pseudoalteromonas tunicata* . Applied and Environmental Microbiology 68: 372–378. 11772647 10.1128/AEM.68.1.372-378.2002 PMC126587

[20] Ausubel F, Brent R, Kingston R, Moore D, Seidman J, et al.. (1990) Current Protocols in Molecular Biology, Vol. 1. Greene.

[21] Church GM, Gilbert W (1984) Genomic sequencing. Proceedings of the National Academy of Sciences 81: 1991–1995. 10.1073/pnas.81.7.1991 PMC345422 6326095

[22] Amann RI, Binder BJ, Olson RJ, Chisholm SW, Devereux R, et al (1990) Combination of 16S rRNA-targeted oligonucleotide probes with flow cytometry for analyzing mixed microbial populations. Applied and Environmental Microbiology 56: 1919–1925. 2200342 10.1128/aem.56.6.1919-1925.1990 PMC184531

[23] Zischler H, Nanda I, Schäfer R, Schmid M, Epplen JT (1989) Digoxigenated oligonucleotide probes specific for simple repeats in DNA fingerprinting and hybridization *in situ* . Human Genetics 82: 227–233. 2731934 10.1007/BF00291160

[24] Bell TA, Lightner DV (1988) A handbook of normal penaeid shrimp histology. World Aquaculture Society. 114 p.

[25] Hayat MA (1993) Stains and cytochemical methods. Plenum Press, New York. 63–64.

[26] Streit A, Stern CD (2001) Combined Whole-Mount *in situ* Hybridization and Immunohistochemistry in Avian Embryos Methods. 23: 339–344. 10.1006/meth.2000.1146 11316435

[27] Harris JO, Burke CM, Maguire GB, et al (1998) Characterization of the digestive tract of greenlip abalone, *Haliotis laevigata* Donovan. I. Morphology and histology. Journal of Shellfish Research 17: 979–988.

[28] Sawabe T, Hayashi K, Moriwaki J, Fukui Y, Thompson FL, et al (2004) *Vibrio neonatus* sp. nov. and *Vibrio ezurae* sp. nov. isolated from the gut of Japanese abalones. Systematic and Applied Microbiology 27: 527–534. 15490553 10.1078/0723202041748154

[29] Stretton S, Techkarnjanaruk S, Mclennan AM, Goodman AE (1998) Use of green fluorescent protein to tag and investigate gene expression in marine bacteria. Appl Environ Microbiol 64: 2554–2559. 9647829 10.1128/aem.64.7.2554-2559.1998 PMC106425

[30] Simon R, Priefer U, Pühler A (1983) A broad host range mobilization system for *in vivo* genetic engineering: transposon mutagenesis in Gram negative bacteria. Nature Biotechnology 1: 784–791.

[31] Yanisch-Perron C, Vieira J, Messing J (1985) Improved M13 phage cloning vectors and host strains: nucleotide sequences of the M13mpl8 and pUC19 vectors. Gene 33: 103–119. 2985470 10.1016/0378-1119(85)90120-9

